# In Toe-Toe

**Published:** 1869-09

**Authors:** 


### IN TOE-TOE.

The "American Journal's" toe is worse—that is, it whines louder. And we have still stronger evidence that its editor is our old schoolmate, Charlie * * , under an assumed name. When a new scholar made his appearance, Charlie always maintained that he came to school only to step on his toe, especially if he had shoes on. Charlie was intensely in earnest about that toe; and when we laughed at him, he thought each one did it "to conceal his vexation," because we had to "toe the mark" and he hadn't. The proof of identy is still stronger: Charlie always tried to tell the truth; so does the "Journal." Charlie didn't always succeed, neither does the "Journal." For example : it speaks of a foot-note we " took the liberty to add to Dr. Morgan's article as published in the REGISTER," when the truth is, we took no liberty at all, but asked Dr. M.'s consent, which was not only cordially granted, but he even suggested an improvement in our language, which was at once adopted, rendering the foot-note more emphatic. The facts are about these: An impertinent puppy, at the Chicago meeting, catechized Dr. M. in reference to his politics, as a test of his fitness for office. Dr. M. supposed the nominating committee sent him, and said so in his article. An editor of the REGISTER was Chairman of the committee, and presided at all its meetings. Knowing that the supposition was not correct, he did not feel willing that the reputation of the whole committee should suffer for the misconduct of one unmannerly dolt, and asked the privilege of a foot-note. And now, the " Journal " (or Charlie, as the case may be) says, " We professed to give our readers Dr. Morgan's statement, and do not admit we were under any obligation to add the comments " W " was pleased to make concerning it, the more so as we regard Dr. M.'s word to be as reliable as that of " W.'s"

But the " Journal " at the same time professed to publish an article from the REGISTER, and mutilated it. Seeing the article credited to the REGISTER,—remembering that one of its editors was chairman of the committee, and knowing that they would not likely keep quiet under a charge of dishonorable conduct, the readers of the " Journal " would naturally conclude that the nominating committee sent that specimen of animated impudence to insult the whole profession of the South, in the person of its most honorable and influential member ; and the " Journal " did all it could do to persuade its readers that all this was true, *when it knew that it was not true.* And there is no occasion to compare Dr. M. and " W " as to veracity; for there has been no conflict. Dr. M. *supposed* " W " as Chairman of the committee *knew*; and Dr. M. appeared gratified to find the facts more creditable than he had supposed.

But Charlie—(excuse us) the " Journal " thinks the REGISTER is opposed to " the formation of a Southern Dental Association." Of course it is ! The REGISTER is opposed to all Dental Associations. It " can not forgive the old Mississippi Valley Association for starting it, and expects to borrow all of poor Job's curses to hurl back in its mother's face one of these days. It publishes a list of the societies once a month only to make fun of them. Its editors have always set their faces like flints against the formation of societies; and that is the reason they never attend any of their meetings.

Our mothers taught us that Charlie's blood was poisoned by the matter from his sore toe, and that we mustn't think hard of him if he talked nonsense and acted ugly; and we didn't, and will not now. When Charlie's whining became unbearable, his mother used to apply a slippery elm poultice to his toe. The "Journal's" mother might get the bark, and have it ready.

